# The relevance of the International Pharmaceutical Federation Global Competency Framework in developing a country-level competency framework for pharmacists: A cross-sectional study

**DOI:** 10.1016/j.rcsop.2021.100095

**Published:** 2021-12-02

**Authors:** Salihah Alfaifi, Naoko Arakawa, Stephanie Bridges

**Affiliations:** a School of Pharmacy, University of Nottingham, University Park, Nottingham NG7 2RD, United Kingdom; b College of Pharmacy, Prince Sattam Bin Abdulaziz University, Alkharj, Saudi Arabia

**Keywords:** Competency, Competency framework, Global competency framework, Pharmacy, Saudi Arabia

## Abstract

**Background:**

In the ever-changing roles of pharmacists, the evidence shows that the use of competency frameworks could aid in achieving professional performance development and ensuring a consistent quality pharmacy education. However, there is no national competency framework for pharmacists in Saudi Arabia. This study, therefore, uses an evidence-based method to identify the competencies required to support and facilitate the pharmacists' training and career development.

**Objective:**

To assess pharmacists' perception of the relevance of the International Pharmaceutical Federation (FIP) Global Competency Framework (GbCF v1) to their own practice.

**Methods:**

A cross-sectional online survey of pharmacists in different practice settings was conducted between August and November 2020, in Saudi Arabia. The survey was adopted from the GbCF v1. A combination of purposive and snowball sampling was used. The relevance to the GbCF v1 was assessed using a four-point Likert scale. Data were analysed using descriptive and inferential statistics.

**Results:**

A total of 522 pharmacists participated in the survey. The study showed broad agreement on relevance to practice for 84% of behaviours included in the GbCF v1. The ‘pharmaceutical public health’ cluster scored the highest percentage of relevant responses (91.42%), followed by the ‘professional/personal’ cluster (87.08%), whereas the ‘organisation and management’ cluster scored the highest percentage of ‘not-relevant’ responses (18.40%). The observed non-relevancy was associated with gender, nationality and area of pharmacy practice (*p* < 0.05).

**Conclusion:**

The competencies and behaviours included in the GbCF v1 are relevant to pharmacy practice in Saudi Arabia. However, some behaviours of the GbCF v1 require modification to be appropriate for the local needs of the Saudi pharmacy practice. The findings from this exercise will be used as a base to develop a foundation-level competency framework to inform initial pharmacy education development and address knowledge gaps and learning needs required to attain and maintain pharmacists' competence to practise.

## 1 Introduction

Pharmacists are critical human resources for health.^1^ Their key role within the health system, particularly in optimising safe, responsible and effective use of medicines, underpins the demand for a highly skilled and competent workforce.^2^^,^^3^ To fulfil their roles, pharmacists must attain and maintain essential competencies relevant to the population health needs in order to ensure a high standard of patient care delivery, thereby helping to improve the health of individual patients and the wider population. Thus, it is crucial to identify the competencies required for pharmacists to enable them to work effectively in the ever-evolving healthcare system.

A competency is defined as a subset of knowledge, skills, attitudes and personal qualities essential to the practice of the healthcare profession at the desired level.^4^ The importance and usefulness of competencies in the professional development of pharmacists has prompted the International Pharmaceutical Federation (FIP) to identify and clarify the competencies required for pharmacists globally to perform their tasks safely, effectively and efficiently.^5^ Each competency comprises a set of behavioural statements that ‘define how that competency would be observed or recognised’.^6^ Therefore, a competency framework typically contains a set of competencies and associated behaviours required for effective performance in practice.^7^

Developing a national competency framework across all sectors can be used as a mapping tool to inform and ensure a consistent quality pharmacy education as well as the professional development of pharmacists.^7^ Evidence shows that using competency frameworks can facilitate professional performance development, identify knowledge gaps and tailor learning needs to maintain and attain fitness to practise.^8^, ^9^, ^10^, ^11^

In 2012, the FIP developed an evidence-based Global Competency Framework version 1 (GbCF v1) to support the foundation level (or early career) practitioners' development worldwide.^12^ The aim of this was to facilitate a seamless transition from initial education through foundation level (day one of registration) to advanced and specialised practice.^2^^,^^13^ The FIP GbCF v1 includes a set of competencies that can be used as a ‘mapping tool’ to develop country-specific frameworks according to the local needs of pharmacy practice. Since its development, it has been used successfully to develop country-specific frameworks for in-service pharmacists as well as pre-service education in a number of countries.14., 15, 16., 17, 18 This provides evidence that the FIP GbCF can be adapted as necessary to relevant for the specific needs of a country. In response to the evolving roles of pharmacists and technology and therapeutic advances, in September 2020, the FIP published the GbCF v2, a revised and updated framework that adds new competencies to reflect these developments.^19^

In Saudi Arabia, there are about 27,000 pharmacists distributed over private and governmental pharmacy sectors.^20^ About 64.7% of pharmacists work in community pharmacies, 31.1% in hospitals, 2.9% in primary healthcare clinics and 0.7% in other settings, including factories, scientific offices and drug stores. 64.8% of the pharmacists are non- Saudi and 81% are males.

In community pharmacies and primary healthcare clinics, pharmacists take on the traditional role of medication dispensing and counselling. In hospitals, pharmacists are responsible for a range of activities, such as compounding and dispensing of medicines, management of medicines storage and supply, provision of drug information as well as pharmaceutical care services.^21^, ^22^, ^23^ In order to practise, pharmacists must complete a Doctor of Pharmacy (PharmD) or a Bachelor's degree in Pharmaceutical Sciences (BPharm) from a higher education institution, sit a national licensure exam and acquire a specific number of hours of continuing pharmaceutical education annually for re-licensing.^24^^,^^25^

No national competency framework or other standards for practice exist for pharmacists in any sector or stage of practice in Saudi Arabia. Although the National Commission for Academic Accreditation and Assessment (NCAAA), the national accrediting institute for higher education in Saudi Arabia, has published a detailed description of learning outcomes for both PharmD and BPharm, no competencies are identified.^26^ However, given that learning outcomes are often used as a tool to measure how well students demonstrate a particular competency, it is essential to first identify competencies in order to define learning outcomes.^4^^,^^27^ Importantly, identification of competencies will inform the development of competency-based education, a modern educational model that aims to instil in health graduates the competencies required to provide care services that meet societal healthcare needs.^28^^,^^29^

The lack of a competency framework raises questions about the quality of pharmacy education, licensure and re-licensure processes to maintain pharmacists' competence and fitness to practise. In addition, there is a consensus of pharmacy experts in Saudi Arabia about the need to improve the current educational outcomes measure of pharmacy programmes at the national level, to ensure that the graduates meet the expected needs of the country.^30^ Hence, there is a need for an evidence-based professional development tool to inform the pharmacy education development process and support current pharmacists to evaluate their capabilities and learning needs for a successful professional development journey.

Therefore, this study aimed to examine the relevance of the FIP GbCF v1 in a Saudi pharmacy practice setting by assessing pharmacists' perceptions of the relevance of the FIP GbCF v1 to their own practice. This will help to identify the competencies required for the effective performance of pharmacists within all sectors of practice, in order to develop a national competency framework for foundation level pharmacists. This will be used to inform and to ensure the quality of initial pharmacy education and, subsequently pharmacy practice in Saudi Arabia.

## 2 Methods

### 2.1 Study design

A cross-sectional anonymous online survey was conducted between August and November 2020. The survey aimed to assess the relevance of the FIP GbCF v1 to the practice of a broad population of pharmacists across different settings of practice in Saudi Arabia. This study was approved by the University of Nottingham's School of Pharmacy Research Ethics Committee (Ref. 011–2019).

### 2.2 Survey development

At the time of the survey development, the second updated version of the FIP GbCF v2 had not been published,^19^ therefore, the survey was fully adopted from the FIP GbCF v1. The survey constituted 100 behavioural statements under 20 competency groups and divided over four broad clusters (Table 1). The respondents were asked to rate the relevance of each statement to their current practice using a four-point Likert scale: highly relevant, relevant, low relevance and not relevant.

**Table 1:** Components of the International Pharmaceutical Federation Global Competency Framework (FIP GbCF v1).

Cluster	Competency group	Number of behaviours within the competency group
Cluster 1: Pharmaceutical public health (PPH)	1. Health promotion	2
	2. Medicines information and advice	2
Cluster 2: Pharmaceutical care (PC)	3. Assessment of medicines	2
	4. Compounding medicines	2
	5. Dispensing	8
	6. Medicines	4
	7. Monitor medicines therapy	3
	8. Patient consultation and diagnosis	6
Cluster 3: Organisation and management (OM)	9. Budget and reimbursement	5
	10. Human resources and management	6
	11. Improvement of service	2
	12. Procurement	7
	13. Supply chain and management	6
	14. Workplace management	6
Cluster 4: Professional/Personal (PP)	15. Communication skills	5
	16. Continuing professional development	8
	17. Legal and regulatory practice	7
	18. Professional and ethical practice	5
	19. Quality assurance and research in workplace	9
	20. Self-management	5

Open-ended questions were added at the end of each cluster where the participants were asked to add any comments or additional behaviours that they consider useful or relevant for each cluster. The online survey was developed using JISC (JISC Online Surveys, Bristol, United Kingdom). It included a cover page providing the background and the purpose of the study, an electronic informed consent form and nine demographic questions. All survey items were designed in English, which is the commonly used language in health professions in Saudi Arabia.

### 2.3 Data collection

All registered pharmacists were eligible for inclusion in this study as foundation level pharmacy practice has not yet been defined in Saudi Arabia. Therefore, a purposive sampling approach was used by approaching the Saudi Commission for Health Specialities (SCFHS), the licensing and registration body for all healthcare providers in Saudi Arabia; and the Saudi Pharmaceutical Society (SPS), the professional society that represents more than 4000 pharmacists in Saudi Arabia, to assist in the survey distribution. They were asked to send the survey anonymous URL link to the emails of all pharmacists included in their registry lists. To increase participation, a snowballing sampling approach was also utilised. The survey URL link was further modified and distributed to the researcher's (SA) contact list via the social media platform, WhatsApp®. Contacts were asked to assist by forwarding the survey URL link to their contacts and colleagues. Following survey distribution, reminders were sent every two weeks for three months.

### 2.4 Data analysis

Quantitative data were downloaded directly into a Microsoft Excel spreadsheet. All data were cleaned and coded manually and systematically checked for errors. Of this, 10% data were cross-checked with the original downloaded Excel spreadsheet. Then, data were exported to the Statistical Package for Social Sciences (SPSS) version 27, for quantitative data analysis using descriptive and inferential statistics. Demographic data were analysed and reported using percentages and frequencies. To ensure a meaningful interpretation of the four-point Likert scale relevance rating, and for the purpose of analysis, it was planned empirically to integrate the response categories of ‘highly relevant’ and ‘relevant’ into one category: relevant; and the response categories of ‘low relevance’ and ‘not relevant’ into one category: not relevant. The agreement on relevance to practice was compared between these categories using percentages and frequencies. The percentages of relevance ratings within each cluster in relation to the respondents' characteristics were represented graphically using heat maps. The cut-off point on relevance to practice was attained when equal to or more than 80% of the responses were in the ‘relevant’ category. The published literature informed the decision to define at least 80% as the cut-off point on relevance rating.^17^^,^^18^^,^^31^^,^^32^ Behavioural statements that had high responses in the ‘not relevant’ category were further analysed using Pearson's Chi-square (χ^2^) test to evaluate the association between the responses and respondents characteristics such as the area of practice and length of time in practice as well as other demographics such as nationality and gender. To identify the significance in the analysis, a probability level of *P* < 0.05 was used. Qualitative data from open-ended questions were downloaded directly into a Microsoft Word document then analysed thematically using Qualitative Analysis Software NVivo version 12.

## 3 Results

### 3.1 Demographics

A total of 522 pharmacists participated in the survey. Of them, 418 (80.1%) were male and 342 (65.5%) non-Saudi. Respondents working in direct patient care (DPC) settings, including community pharmacy, hospital pharmacy and primary healthcare centres, accounted for 79.3% of the sample. Respondents' median length of practice was ten years (IQR ±10). Respondents' characteristics are shown in Table 2.

**Table 2:** Respondents characteristics (*n* = 522).

Median Age (IQR)		37 years (±11)
Gender	Male	418 (80.1%)
	Female	104 (19.9%)
Nationality	Saudi	180 (34.5%)
	Non-Saudi	342 (65.5%)
**Province**	Riyadh	224 (42.9%)
	Makkah	119 (22.8%)
	Eastern province	73 (14%)
	Madinah	19 (3.6%)
	Qassim	13 (2.5%)
	Asir	20 (3.8%)
	Najran	7 (1.3%)
	Bahah	3 (0.6%)
	Hai'l	11 (2.1%)
	Tabuk	6 (1.1%)
	Jawf	5 (1%)
	Northern borders	7 (1.3%)
	Jizan	15 (2.9%)
Initial degree of education	BPharm	357 (68.4%)
	PharmD	69 (13.2%)
Hold any postgraduate education	Yes	124 (23.7%)
Median years qualified (IQR)		11 years (IQR ± 13)
Years qualified	0 to 5 years	137 (26.2)
	6 to 10 years	106 (20.3)
	11 to 20 years	207 (39.7)
	21 to 30 years	56 (10.7)
	>30 years	16 (3.1)
Patient-facing setting	DPC	414 (79.3)
	NDPC	108 (20.7)
Median years in current area of practice (IQR)		10 (IQR ± 10)
Years in current area of practice	≤1 years	38 (7.3)
	2 to 3 years	57 (10.9)
	4 to 5 years	71 (13.6)
	6 to 10 years	122 (23.4)
	11 to 20 years	190 (36.4)
	21 to 30 years	35 (6.7)
	>30 years	9 (1.7)
Practice setting	Community pharmacy	220 (42.1%)
	Hospital	166 (31.8%)
	Primary healthcare centre	28 (5.4%)
	Academia	27 (5.2%)
	Industry	52 (10%)
	Regulatory organisations	20 (3.8%)
	Other	9 (1.7%)
Previous work experience in other practice area	Yes	493 (94.4%)

Abbreviations for respondents characteristics: Interquartile rang (IQR), Bachelor's degree in pharmacy (BPharm), Doctor of pharmacy (PharmD), Direct Patient Care (DPC), Non-Direct Patient Care (NDPC).

### 3.2 Relevance rating—overall

From the 100 behavioural statements rated within the four competency clusters, 84 met the cut-off point of at least 80% agreement over relevance (Table 3). The aggregated responses of each cluster showed that the PPH cluster scored the highest percentage of relevant responses (91.42%), followed by the PP cluster (87.08%), while a higher percentage of not relevant responses were evident in the OM cluster and PC cluster (18.40% and 14.66% respectively).

**Table 3:** Relevance rating of the FIP GbCF v1 behavioural statements.

Competency group	Behavioural statements	Total	Relevant	Not relevant
			Count (%)	Count (%)
Cluster 1: Pharmaceutical Public Health (PPH) competencies				
1. Health promotion	1.1. Assess the primary healthcare needs (taking into account the cultural and social setting of the patient)	522	477 (91.4%)	45 (8.6%)
	1.2. Advise on health promotion, disease prevention and control, and healthy lifestyle	522	478 (91.6%)	44 (8.4%)
2. Medicines information and advice	2.1. Counsel population on the safe and rational use of medicines and devices (including the selection, use, contraindications, storage, and side effects of non-prescription and prescription medicines)	522	485 (92.9%)	37 (7.1%)
	2.2. Identify sources, retrieve, evaluate, organise, assess and disseminate relevant medicines information according to the needs of patients and clients and provide appropriate information	522	469 (89.8%)	53 (10.2%)
**PPH cluster average**		**2088**	**1909 (91.42%)**	**179 (8.5%)**

Cluster 2: Pharmaceutical Care (PC) competencies				
3. Assessment of medicines	3.1. Appropriately select medicines (e.g. according to the patient, hospital, government policy, etc.)	522	470 (90%)	52 (10%)
	3.2. Identify, prioritise and act upon medicine-medicine interactions; medicine-disease interactions; medicine-patient interactions; medicines-food interactions	522	484 (92.7%)	38 (7.3%)
4.Compounding medicines	4.1. Prepare pharmaceutical medicines (e.g. extemporaneous, cytotoxic medicines), determine the requirements for preparation (calculations, appropriate formulation, procedures, raw materials, equipment etc.)	522	386 (73.9%)	136 (26.1%)
	4.2. Compound under the good manufacturing practice for pharmaceutical (GMP) medicines	522	401 (76.8%)	121 (23.2%)
5. Dispensing	5.1. Accurately dispense medicines for prescribed and/or minor ailments and monitor the dispense (re-checking the medicines)	522	473 (90.6%)	49 (9.4%)
	5.2. Accurately report defective or substandard medicines to the appropriate authorities.	522	451 (86.4%)	71 (13.6%)
	5.3. Appropriately validate prescriptions, ensuring that prescriptions are correctly interpreted and legal	522	471 (90.2%)	51 (9.8%)
	5.4. Dispense devices (e.g. inhaler or a blood glucose meter)	522	431 (82.6%)	91 (17.4%)
	5.5. Document and act upon dispensing errors	522	445 (85.2%)	77 (14.8%)
	5.6. Implement and maintain a dispensing error reporting system and a ‘near misses’ reporting system	522	430 (82.4%)	92 (17.6%)
	5.7. Label the medicines (with the required and appropriate information)	522	459 (87.9%)	63 (12.1%)
	5.8. Learn from and act upon previous ‘near misses’ and ‘dispensing errors’	522	447 (85.6%)	75 (14.4%)
6. Medicines	6.1. Advise patients on proper storage conditions of the medicines and ensure that medicines are stored appropriately (e.g. humidity, temperature, expiry date, etc.)	522	477 (91.4%)	45 (8.6%)
	6.2. Appropriately select medicine formulation and concentration for minor ailments (e.g. diarrhoea, constipation, cough, hay fever, insect bites, etc.)	522	471 (90.2%)	51 (9.8%)
	6.3. Ensure appropriate medicines, route, time, dose, documentation, action, form and response for individual patients	522	489 (93.7%)	33 (6.3%)
	6.4. Package medicines to optimise safety (ensuring appropriate re-packaging and labelling of the medicines)	522	451 (86.4%)	71 (13.6%)
7. Monitor medicines therapy	7.1. Apply guidelines, medicines formulary system, protocols and treatment pathways	522	460 (88.1%)	62 (11.9%)
	7.2. Ensure therapeutic medicines monitoring, impact and outcomes (including objective and subjective measures)	522	448 (85.8%)	74 (14.2%)
	7.3. Identify, prioritise and resolve medicines management problems (including errors)	522	455 (87.2%)	67 (12.8%)
8. Patient consultation and diagnosis	8.1. Apply first aid and act upon arranging follow-up care	522	398 (76.2%)	124 (23.8%)
	8.2. Appropriately refer	522	424 (81.2%)	98 (18.8%)
	8.3. Assess and diagnose based on objective and subjective measures	522	415 (79.5%)	107 (20.5%)
	8.4. Discuss and agree with the patients the appropriate use of medicines, taking into account patients' preferences	522	444 (85.1%)	78 (14.9%)
	8.5. Document any intervention (e.g. document allergies, medicines and food, in patient medicines history)	522	431 (82.6%)	91 (17.4%)
	8.6. Obtain, reconcile, review, maintain and update relevant patient medication and diseases history	522	426 (81.6%)	96 (18.4%)
**PC cluster average**		**13,050**	**11,137 (85.34%)**	**1913 (14.66%)**

Cluster 3: Organisation and Management (OM) competencies				
9. Budget and reimbursement	9.1. Acknowledge the organisational structure	522	432 (82.8%)	90 (17.2%)
	9.2. Effectively set and apply budgets	522	404 (77.4%)	118 (22.6%)
	9.3. Ensure appropriate claim for the reimbursement	522	386 (73.9%)	136 (26.1%)
	9.4. Ensure financial transparency	522	391 (74.9%)	131 (25.1%)
	9.5. Ensure proper reference sources for service reimbursement	522	385 (73.8%)	137 (26.2%)
10. Human resources management	10.1. Demonstrate organisational and management skills (e.g. know, understand and lead on medicines management, risk management, self-management, time management, people management, project management, policy management)	522	429 (82.2%)	93 (17.8%)
	10.2. Identify and manage human resources and staffing issues	522	397 (76.1%)	125 (23.9%)
	10.3. Participate, collaborate, advise in therapeutic decision-making and use appropriate referral in a multi-disciplinary team	522	433 (83%)	89 (17%)
	10.4. Recognise and manage the potential of each member of the staff and utilise systems for performance management (e.g. carry out staff appraisals)	522	415 (79.5%)	107 (20.5%)
	10.5. Recognise the value of the pharmacy team and of a multidisciplinary team	522	449 (86%)	73 (14%)
	10.6. Support and facilitate staff training and continuing professional development	522	458 (87.7%)	64 (12.3%)
11. Improvement of service	11.1. Identify and implement new services (according to local needs)	522	447 (85.6%)	75 (14.4%)
	11.2. Resolve, follow up and prevent medicines related problems	522	462 (88.5%)	60 (11.5%)
12. Procurement	12.1. Access reliable information and ensure the most cost-effective medicines in the right quantities with the appropriate quality	522	436 (83.5%)	86 (16.5%)
	12.2. Develop and implement contingency plan for shortages	522	434 (83.1%)	88 (16.9%)
	12.3. Efficiently link procurement to formulary, to push/pull system (supply chain management) and payment mechanisms	522	416 (79.7%)	106 (20.3%)
	12.4. Ensure there is no conflict of interest	522	413 (79.1%)	109 (20.9%)
	12.5. Select reliable supplies of high-quality products (including appropriate selection process, cost effectiveness, timely delivery)	522	422 (80.8%)	100 (19.2%)
	12.6. Supervise procurement activities	522	408 (78.2%)	114 (21.8%)
	12.7. Understand the tendering methods and evaluation of tender bids	522	402 (77%)	120 (23%)
13. Supply chain and management	13.1. Demonstrate knowledge in store medicines to minimise errors and maximise accuracy	522	439 (84.1%)	83 (15.9%)
	13.2. Ensure accurate verification of rolling stocks	522	426 (81.6%)	96 (18.4%)
	13.3. Ensure effective stock management and running of service with the dispensary	522	430 (82.4%)	92 (17.6%)
	13.4. Ensure logistics of delivery and storage	522	418 (80.1%)	104 (19.9%)
	13.5. Implement a system for documentation and record keeping	522	420 (80.5%)	102 (19.5%)
	13.6. Take responsibility for quantification of forecasting	522	420 (80.5%)	102 (19.5%)
14. Workplace management	14.1. Address and manage day to day management issues	522	453 (86.8%)	69 (13.2%)
	14.2. Demonstrate the ability to take accurate and timely decisions and make appropriate judgements	522	456 (87.4%)	66 (12.6%)
	14.3. Ensure the production schedules are appropriately planned and managed	522	433 (83%)	89 (17%)
	14.4. Ensure the work time is appropriately planned and managed	522	453 (86.8%)	69 (13.2%)
	14.5. Improve and manage the provision of pharmaceutical services	522	446 (85.4%)	76 (14.6%)
	14.6. Recognise and manage pharmacy resources (e.g. financial, infrastructure)	522	418 (80.1%)	104 (19.9%)
**OM Cluster average**		**16,704**	**13,631 (81.60%)**	**3073 (18.40%)**

Cluster 4: Professional/ Personal (PP) competencies				
15. Communication skills	15.1. Communicate clearly, precisely and appropriately while being a mentor or tutor	522	496 (95%)	26 (5%)
	15.2. Communicate effectively with health and social care staff, support staff, patients, carer, family relatives and clients/customers, using lay terms and checking understanding	522	489 (93.7%)	33 (6.3%)
	15.3. Demonstrate cultural awareness and sensitivity	522	475 (91%)	47 (9%)
	15.4. Tailor communications to patient needs	522	477 (91.4%)	45 (8.6%)
	15.5. Use appropriate communication skills to build, report and engage with patients, health and social care staff and voluntary services (e.g. verbal and non-verbal)	522	479 (91.8%)	43 (8.2%)
16. Continuing Professional Development (CPD)	16.1. Document CPD activities	522	454 (87%)	68 (13%)
	16.2. Engage with students/interns/residents	522	442 (84.7%)	80 (15.3%)
	16.3. Evaluate currency of knowledge and skills	522	459 (87.9%)	63 (12.1%)
	16.4. Evaluate learning	522	448 (85.8%)	74 (14.2%)
	16.5. Identify if expertise needed outside the scope of knowledge	522	443 (84.9%)	79 (15.1%)
	16.6. Identify learning needs	522	446 (85.4%)	76 (14.6%)
	16.7. Recognise own limitations and act upon them	522	455 (87.2%)	67 (12.8%)
	16.8. Reflect on performance	522	465 (89.1%)	57 (10.9%)
17. Legal and regulatory practice	17.1. Apply and understand regulatory affairs and the key aspects of pharmaceutical registration and legislation	522	461 (88.3%)	61 (11.7%)
	17.2. Apply knowledge in relation to the principals of business economics and intellectual property rights including the basics of patent interpretation	522	438 (83.9%)	84 (16.1%)
	17.3. Be aware of and identify the new medicines coming to the market	522	461 (88.3%)	61 (11.7%)
	17.4. Comply with legislation for drugs with the potential for abuse	522	456 (87.4%)	66 (12.6%)
	17.5. Demonstrate knowledge in marketing and sales	522	434 (83.1%)	88 (16.9%)
	17.6. Engage with health and medicines policies	522	468 (89.7%)	54 (10.3%)
	17.7. Understand the steps needed to bring a medicinal product to the market including the safety, quality, efficacy and pharmacoeconomic assessments of the product	522	451 (86.4%)	71 (13.6%)
18. Professional and ethical practice	18.1. Demonstrate awareness of local /national codes of ethics	522	474 (90.8%)	48 (9.2%)
	18.2. Ensure confidentiality (with the patient and other healthcare professionals)	522	484 (92.7%)	38 (7.3%)
	18.3. Obtain patient consent (it can be implicit on occasion)	522	450 (86.2%)	72 (13.8%)
	18.4. Recognise own professional limitations	522	473 (90.6%)	49 (9.4%)
	18.5. Take responsibility for own action and for patient care	522	469 (89.8%)	53 (10.2%)
19. Quality assurance and research in the workplace	19.1. Apply research findings and understand the benefit risk (e.g. pre-clinical, clinical trials, experimental clinical-pharmacological research and risk management)	522	415 (79.5%)	107 (20.5%)
	19.2. Audit quality of service (ensure that they meet local and national standards and specifications)	522	432 (82.8%)	90 (17.2%)
	19.3. Develop and implement Standing Operating Procedures (SOPs)	522	427 (81.8%)	95 (18.2%)
	19.4. Ensure appropriate quality control tests are performed and managed appropriately	522	417 (79.9%)	105 (20.1%)
	19.5. Ensure medicines are not counterfeit and quality standards	522	436 (83.5%)	86 (16.5%)
	19.6. Identify and evaluate evidence-base to improve the use of medicines and services	522	433 (83%)	89 (17%)
	19.7. Identify, investigate, conduct, supervise and support research at workplace (enquiry-driven practice)	522	421 (80.7%)	101 (19.3%)
	19.8. Implement, conduct and maintain a reporting system of pharmacovigilance (e.g. report Adverse Drug Reactions)	522	437 (83.7%)	85 (16.3%)
	9.9. Initiate and implement audit and research activities	522	422 (80.8%)	100 (19.2%)
20. Self-management	20.1. Apply assertiveness skills (inspire confidence)	522	476 (91.2%)	46 (8.8%)
	20.2. Demonstrate leadership and practice management skills, initiative and efficiency	522	476 (91.2%)	46 (8.8%)
	20.3. Document risk management (e.g. critical incidents)	522	453 (86.8%)	69 (13.2%)
	20.4. Ensure punctuality	522	467 (89.5%)	55 (10.5%)
	20.5. Prioritise work and implement innovative ideas	522	470 (90%)	52 (10%)
**PP cluster average**		**20,358**	**17,729 (87.1%)**	**2629 (12.9%)**

### 3.3 Relevance rating—per area of practice

**Fig. 1:**
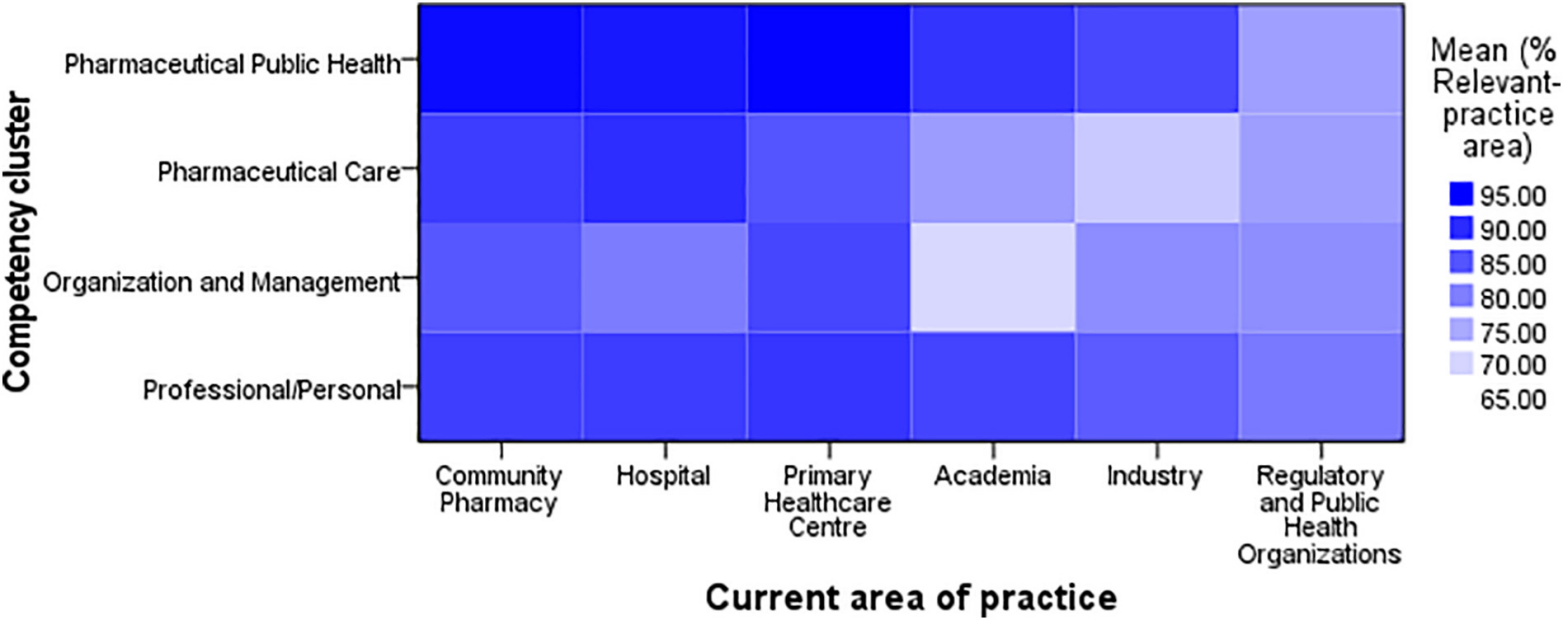
Heat map showing relevance rating between the respondents' areas of practice.

Respondents from DPC settings, including community pharmacy, hospital and primary health care centres, showed a higher relevance across all clusters compared to Non-Direct Patient Care (NDPC) settings, including academia, industry and regulatory organisations (Fig. 1). Academia and industry showed little engagement in OM (academia = 69.56%; industry = 78.36%) and PC (academia = 76.59%; industry = 71.38%) compared to PPH (academia = 88.88%; industry = 86.53%) and PP (academia = 86.98%; industry = 84.31%) clusters.

### 3.4 Relevance rating—per length working in current area of practice

**Fig. 2:**
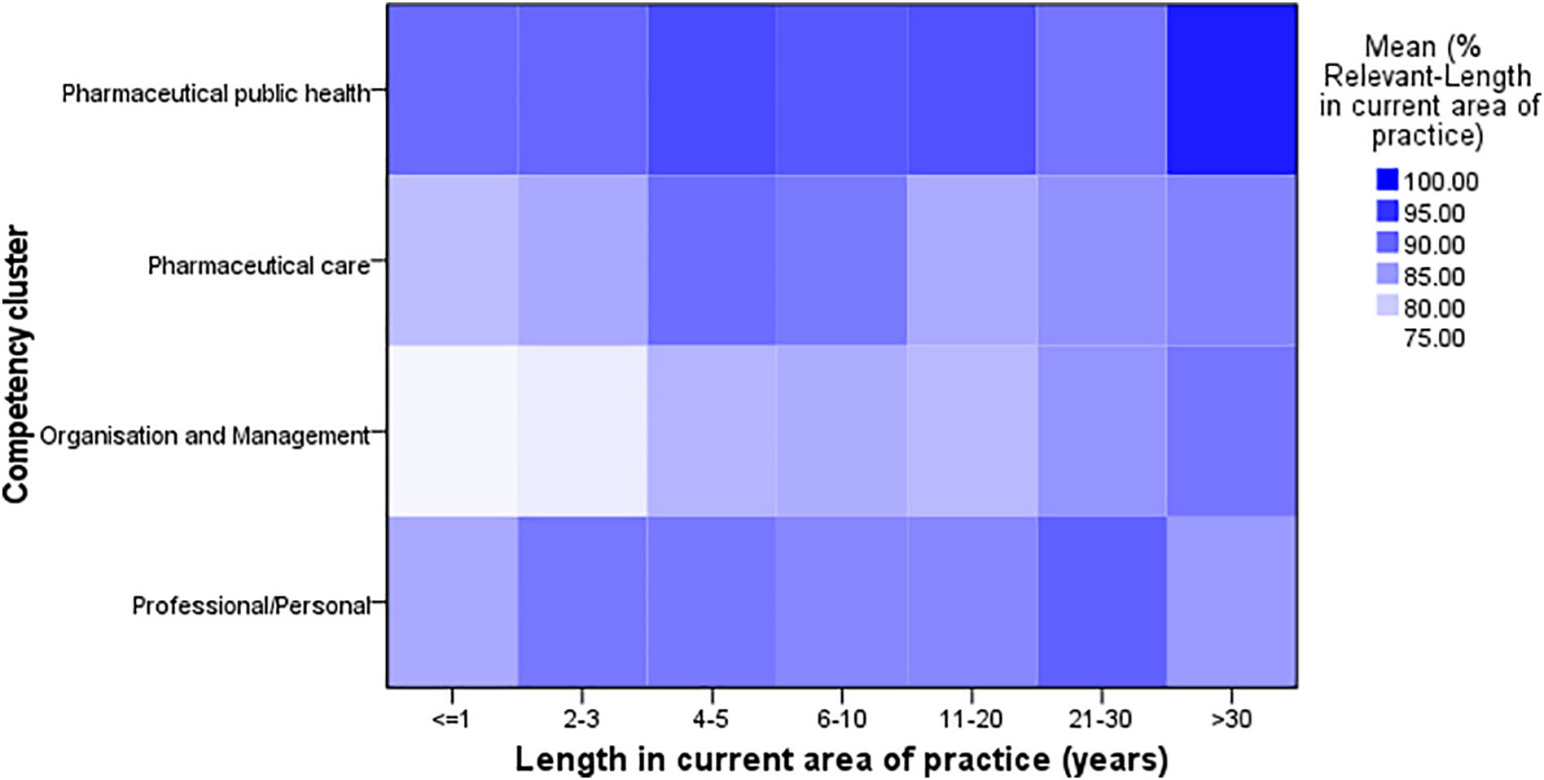
Heat map showing relevance rating between the respondents' years in current area of practice.

Fig. 2 shows a comparison between years working in current area of practice in relation to each competency cluster. Data of PC cluster show steady progress from early years until 10 years followed by a slight progress in the following years(≤1 years = 81.36%, 2–3 years = 83.43%, 4–5 years = 89.29%, 6–10 years = 88.03%, 11–20 = 83.34%, 21–30 years = 85.71%, >30 years = 87.11%). However, data from OM cluster show slight progress over years (≤1 years = 75.90%, 2–3 years = 76.86%, 4–5 years = 82.35%, 6–10 years = 82.94%, 11–20 = 81.99%, 21–30 years = 85.44%, >30 years = 88.54%). Data from PPH and PP clusters show a steady and a gradual increase over years.

### 3.5 Relevance rating—per nationality

Table 4 presents a comparison between respondents' nationality with each competency cluster. Data showed that non-Saudi respondents have showed a higher relevance to all clusters compared to Saudi respondents: PPH (94.59%, 85.41%, respectively), PC (87.15%, 81.88%, respectively), OM (85.20%, 74.75%, respectively) and PP (88.80, 83.81%, respectively).

**Table 4:** Percentage of ‘relevant’ responses in each cluster between Saudi and non-Saudi respondents.

Clusters	Nationality (% N-‘relevant’)	
	Saudi	Non-Saudi
Pharmaceutical public health (PPH)	85.41	94.59
Pharmaceutical Cate (PC)	81.88	87.15
Organisation and Management (OM)	74.75	85.20
Professional/Personal (PP)	83.81	88.80

### 3.6 Relevance rating- per gender

Table 5 presents a comparison between the gender of respondents and each competency cluster. Data showed an overall high relevance to all clusters in favour of male respondents than female respondents. Data of PPH and OM, in particular, showed high relevance of male respondents (92.34%, 83.62%, respectively) compared to female respondents (87.74%, 73.49%, respectively).

**Table 5:** Percentage of ‘relevant’ responses in each cluster between male and female respondents.

Clusters	Gender (% N-‘relevant’)	
	Male	Female
Pharmaceutical public health (PPH)	92.34	87.74
Pharmaceutical Cate (PC)	85.96	82.84
Organisation and Management (OM)	83.62	73.49
Professional/Personal (PP)	87.58	85.08

### 3.7 Responses from open-ended questions

A total of 121 open-ended responses were received from the survey respondents: 52 responses were within the PPH cluster, 27 within the PC cluster, 25 within the OM cluster and 17 within the PP cluster. The responses were analysed thematically and grouped into 36 behaviours. The identified behaviours were then mapped against behaviours of GbCF V1 and V2. Out of 36 behaviours: 23 matched the FIP GbCF v1, 11 matched the FIP GbCF v2, and 2 new behaviours match neither. The new behaviours are: provide point of care testing in community pharmacy for patients and community members; and discuss and agree with the patients about the appropriate generic substitution of medications according to patients' preference and or budget.

### 3.8 Relevance ratings—areas of disagreement overall

Findings shows that 16 behavioural statements have a percentage above the predefined cut-off point of relevance (20%) from the PC, OM and PP clusters. These behaviours represent two from ‘compounding medicines’ competency group, two from ‘patient consultation and diagnosis’, four from ‘budget and reimbursement’, two from ‘human resources and management’, four from ‘procurement’ and two from ‘quality assurance and research in workplace’ (Table 3).

### 3.9 Areas of disagreement—per area of practice

A Pearson's Chi-square (χ^2^) test showed that eight out of the 16 behavioural statements of disagreement over relevancy were associated with the area of pharmacy practice (*P* < 0.05) (Table 6). A higher percentage of respondents working in community pharmacy were more likely to perceive behaviours in the PC (4.1, 4.2, 8.1, 8.3) and the OM (10.2, 12.7) as being ‘not relevant’ to their practice compared to respondents working in other practice settings. Respondents working in hospitals were more likely (compared to other settings) to perceive behaviour in the OM (9.4, 12.6) as being ‘not relevant’.

**Table 6:** Distribution of the ‘not relevant’ responses for the 16 behaviours of disagreement in relation to area of practice.

Behavioural statements of disagreement		Area of pharmacy practice (% N ‘not-relevant’)							χ^2^-value *P*-value
		Community pharmacy	Hospital	Primary healthcare clinics	Academia	Industry	Regulatory organisations	Other	
**Cluster2: Pharmaceutical care (PC) competencies**									
**4.Compounding medicine competency group**	4.1 Prepare pharmaceutical medicines (e.g. extemporaneous, cytotoxic medicines), determine the requirements for preparation (calculations, appropriate formulation, procedures, raw materials, equipment etc.)	48.5	14.7	8.1	6.6	15.4	4.4	2.2	χ^2^ = 27.919 *P* < 0.001
	4.2. Compound under the good manufacturing practice for pharmaceutical (GMP) medicines	40.5	22.3	8.3	5.8	14.9	4.1	4.1	χ^2^ = 16.298 *P* = 0.012
**8. Patient consultation and diagnosis competency group**	8.1. Apply first aid and act upon arranging follow-up care	31.5	30.6	4	7.3	15.3	5.6	5.6	χ^2^ = 26.977 P < 0.001
	8.3. Assess and diagnose based on objective and subjective measures	31.8	29	4.7	8.4	16.8	5.6	3.7	χ^2^ = 17.256 *P* = 0.008

**Cluster 3: Organisation and management (OM) competencies**									
**9. Budget and reimbursement competency group**	9.2. Effectively set and apply budgets	35.6	39.8	5.1	6.8	7.6	3.4	1.7	χ^2^ = 6.341 *P* = 0.386
	9.3. Ensure appropriate claim for the reimbursement	34.6	39.7	3.7	7.4	9.6	3.7	1.5	χ^2^ = 8.903 *P* = 0.179
	9.4. Ensure financial transparency	35.1	37.4	3.1	9.9	8.4	3.1	3.1	χ^2^ = 15.650 *P* = 0.016
	9.5. Ensure proper reference sources for service reimbursement	33.6	37.2	5.8	8.8	8.8	3.6	2.2	χ^2^ = 10.173 *P* = 0.118
**10. Human resources and management competency group**	10.2. Identify and manage human resources and staffing issues	34.4	29.6	4.8	9.6	13.6	6.4	1.6	χ^2^ = 13.946 *P* = 0.030
	10.4. Recognise and manage the potential of each member of the staff and utilise systems for performance management (e.g. carry out staff appraisals)	36.4	30.8	6.5	8.4	11.2	5.6	0.9	χ^2^ = 5.960 *P* = 0.428
**12.Procurement competency group**	12.3. Efficiently link procurement to formulary, to push/pull system (supply chain management) and payment mechanisms	29.2	36.8	5.7	9.4	11.3	4.7	2.8	χ^2^ = 12.449 *P* = 0.053
	12.4. Ensure there is no conflict of interest	40.4	31.2	2.8	9.2	9.2	3.7	3.7	χ^2^ = 9.257 *P* = 0.160
	12.6. Supervise procurement activities	32.5	33.3	3.5	10.5	12.3	4.4	3.5	χ^2^ = 15.974 *P* = 0.014
	12.7. Understand the tendering methods and evaluation of tender bids	35.8	33.3	4.2	10.8	8.3	5.8	1.7	χ^2^ = 13.706 *P* = 0.033

**Cluster 4: Professional/ Personal (PP) competencies**									
**19. Quality assurance and research in workplace competency group**	19.1. Apply research findings and understand the benefit risk (e.g. pre-clinical, clinical trials, experimental clinical-pharmacological research and risk management)	42.1	29.9	6.5	3.7	12.1	4.7	0.9	χ^2^ = 2.418 *P* = 0.878
	19.4. Ensure appropriate quality control tests are performed and managed appropriately	42.9	22.9	7.6	4.8	14.3	5.7	1.9	χ^2^ = 8.319 *P* = 0.216

### 3.10 Areas of disagreement—per nationality and gender

Table 7 and Table 8 showed that non-Saudi and male respondents were more likely to perceive behaviours in the OM (9.2, 9.3, 9.4, 9.5, 10.2, 10.4, 12.6, 12.7) as ‘not relevant’ to their practice (*P* < 0.05). Table 7 showed also that Non-Saudi respondents were more likely to perceive behaviour 8.1 and 8.3 from the PC as ‘not-relevant’ to their practice (P < 0.05). Moreover, Table 8 showed that male respondents were more likely to perceive behaviour 19.1 in the PP as not relevant (P < 0.05).

**Table 7:** Distribution of the ‘not relevant’ responses for the 16 behaviours of disagreement in relation to nationality.

Behavioural statements of disagreement		Nationality (% N ‘not-relevant’)		χ^2^- value p-value
		Saudi	Non-Saudi	
**Cluster 2: Pharmaceutical Care (PC) competencies**				
**4. Compounding medicines competency group**	4.1. Prepare pharmaceutical medicines (e.g. extemporaneous, cytotoxic medicines), determine the requirements for preparation (calculations, appropriate formulation, procedures, raw materials, equipment etc.)	28.7	71.3	χ^2^ = 2.744 *P* = 0.098
	4.2. Compound under the good manufacturing practice for pharmaceutical (GMP) medicines	36.4	63.6	χ^2^ = 0.247 *P* = 0.619
**8. Patient consultation and diagnosis competency group**	8.1. Apply first aid and act upon arranging follow-up care	45.2	54.8	χ^2^ = 8.209 *P* = 0.004
	8.3. Assess and diagnose based on objective and subjective measures	47.7	52.3	χ^2^ = 10.350 *P* = 0.001

**Cluster 3: Organisation and Management (OM) competencies**				
**9. Budget and reimbursement competency group**	9.2. Effectively set and apply budgets	44.1	55.9	χ^2^ = 6.200 *P* = 0.013
	9.3. Ensure appropriate claim for the reimbursement	43.4	56.6	χ^2^ = 6.448 *P* = 0.011
	9.4. Ensure financial transparency	47.3	52.7	χ^2^ = 12.773 P < 0.001
	9.5. Ensure proper reference sources for service reimbursement	46.7	53.3	χ^2^ = 12.303 P < 0.001
**10. Human resources and management competency group**	10.2. Identify and manage human resources and staffing issues	44.8	55.2	χ^2^ = 7.744 *P* = 0.005
	10.4. Recognise and manage the potential of each member of the staff and utilise systems for performance management (e.g. carry out staff appraisals)	45.8	54.2	χ^2^ = 7.623 *P* = 0.006
**12. Procurement competency group**	12.3. Efficiently link procurement to formulary, to push/pull system (supply chain management) and payment mechanisms	50.9	49.1	χ^2^ = 15.952 P < 0.001
	12.4. Ensure there is no conflict of interest	45	55	χ^2^ = 6.686 *P* = 0.010
	12.6. Supervise procurement activities	49.1	50.9	χ^2^ = 13.837 P < 0.001
	12.7. Understand the tendering methods and evaluation of tender bids	45.8	54.2	χ^2^ = 8.886 *P* = 0.003

**Cluster 4: Professional/Personal competencies**				
**19. Quality assurance and research in workplace competency group**	19.1. Apply research findings and understand the benefit risk (e.g. pre-clinical, clinical trials, experimental clinical-pharmacological research and risk management)	42.1	57.9	χ^2^ = 3.417 *P* = 0.065
	19.4. Ensure appropriate quality control tests are performed and managed appropriately	41	59	χ^2^ = 2.435 *P* = 0.119

**Table 8:** distribution of the ‘not relevant’ responses for the 16 behaviours of disagreement in relation to gender.

Behavioural statements of disagreement		Gender (% N ‘not-relevant’)		χ^2^- value p-value
		Male	Female	
**Cluster 2: Pharmaceutical Care (PC) competencies**				
**4. Compounding medicines competency group**	4.1. Prepare pharmaceutical medicines (e.g. extemporaneous, cytotoxic medicines), determine the requirements for preparation (calculations, appropriate formulation, procedures, raw materials, equipment etc.)	84.6	15.4	χ^2^ = 2.316 *P* = 0.128
	4.2. Compound under the good manufacturing practice for pharmaceutical (GMP) medicines	81	19	χ^2^ = 0.083 *P* = 0.774
**8. Patient consultation and diagnosis**	8.1. Apply first aid and act upon arranging follow-up care	75	25	χ^2^ = 2.627 *P* = 0.105
	8.3. Assess and diagnose based on objective and subjective measures	74.8	25.2	χ^2^ = 2.379 *P* = 0.123

**Cluster 3: Organisation and Management (OM) competencies**				
**9. Budget and reimbursement competency group**	9.2. Effectively set and apply budgets	72.9	27.1	χ^2^ = 4.948 *P* = 0.026
	9.3. Ensure appropriate claim for the reimbursement	72.8	27.2	χ^2^ = 6.114 P = 0.013
	9.4. Ensure financial transparency	72.5	27.5	χ^2^ = 6.261 P = 0.012
	9.5. Ensure proper reference sources for service reimbursement	70.1	29.9	χ^2^ = 11.651 P = 0.001
**10. human resources and management competency group**	10.2. Identify and manage human resources and staffing issues	73.6	26.4	χ^2^ = 4.321 *P* = 0.038
	10.4. Recognise and manage the potential of each member of the staff and utilise systems for performance management (e.g. carry out staff appraisals)	69.2	30.8	χ^2^ = 10.056 *P* = 0.002
**12. Procurement competency group**	12.3. Efficiently link procurement to formulary, to push/pull system (supply chain management) and payment mechanisms	67.9	32.1	χ^2^ = 12.312 P < 0.001
	12.4. Ensure there is no conflict of interest	73.4	26.6	χ^2^ = 3.856 *P* = 0.050
	12.6. Supervise procurement activities	71.9	28.1	χ^2^ = 6.068 P = 0.014
	12.7. Understand the tendering methods and evaluation of tender bids	70.8	29.2	χ^2^ = 8.345 P = 0.004

**Cluster 4: Professional/Personal (PP) competencies**				
**19. Quality assurance and research in workplace competency group**	19.1. Apply research findings and understand the benefit risk (e.g. pre-clinical, clinical trials, experimental clinical-pharmacological research and risk management)	72	28	χ^2^ = 5.554 *P* = 0.018
	19.4. Ensure appropriate quality control tests are performed and managed appropriately	76.2	23.8	χ^2^ = 1.244 *P* = 0.265

## 4 Discussion

To our knowledge, this is the first study to explore the relevance of the FIP GbCF in Saudi pharmacy practice. This national survey investigated the relevance of the FIP GbCF v1 to the practice of pharmacists from different practice settings: community pharmacy, hospitals, primary health care clinics, pharmaceutical industry, academia and others.

The survey shows a broad agreement on relevance to practice for 84% of behaviours included in the GbCF v1. The PPH cluster scored the highest percentage of relevant responses (91.42%), followed by the PP cluster (87.08%) and the PC cluster (85.34%). On the other hand, the OM cluster scored the lowest percentage of relevant responses by the study population (81.60%). These findings are consistent with other global studies, which have shown similar findings on relevancy to the FIP GbCF.^10^^,^^18^^,^^32^

In this study, the level of the relevance of OM competencies was low since pharmacists receive little or no training on these competencies in either undergraduate or post- graduate education.^33^^,^^34^ This lack raises concerns about pharmacists' competencies to apply these skills in their practice especially when they undertake management roles. Alarifi (2013) reported that community pharmacists mostly acquire their managerial skills on the job and that few had post-graduate training in management or leadership.^35^ The author suggests that the lack of formalised training programmes contributes to the difference in skills across the study population. Hence, incorporating these competencies at foundation level is highly warranted to enable pharmacists' transition to leadership roles and ensure a consistently high calibre of professional performance.

Respondents from DPC settings were more likely to perceive behaviours included in the FIP GbCF as relevant to their practice compared to respondents from the NDPC settings. This might be related to their scope of practice, which involves more responsibilities within health promotion, medicines compounding, dispensing and procurement activities.^22^ The low relevance noted in ‘budget and reimbursement, ‘human resources management’ and ‘procurement’ competency groups might be related to the fact that these responsibilities are conducted at the level of organisation or sector rather than on departmental level. Respondents rated low relevance for ‘patient consultation and diagnosis' behaviours which might be explained in that patient diagnosis and assessment is not a common nor a regulated practice for pharmacists in Saudi Arabia.^21^^,^^22^ Interestingly, recent local research reported that the provision of such services in community pharmacies is increasingly desired by consumers.^36^, ^37^, ^38^

Respondents also scored low relevance for ‘quality assurance and research in workplace’ behaviours. This is consistent with AlRuthia et al. (2018) finding that pharmacists' participation in pharmacy practice research is limited, despite a high proportion working in DPC roles.^39^ Most pharmacy practice research is lead mainly, not by practising pharmacists, but by researchers who are away from practice, including academics with claims that this is primarily to achieve academic promotion or by students to fulfil their graduation requirements.^22^ This finding raises concerns about pharmacists' research skills and the presence of a research supporting environment, time and resources for practising pharmacists in Saudi Arabia. Interestingly, these findings are broadly similar to the situation Alhaqan et al. (2020) reported in Kuwait,^18^ a neighbouring country with similar pattern and scope of pharmacy practice.

While the area of practice was reported previously as a factor influencing pharmacists' perception of the FIP GbCF items relevancy,^10^^,^^18^^,^^31^ this study adds to the increasing body of knowledge that the nationality and gender of pharmacists may influence their perceptions of the behaviours included in the FIP GbCF. Of the 16 ‘not relevant’ behaviours, 12 and 10 of them were associated with nationality and gender, respectively.

Table 4 shows that non-Saudi pharmacists perceived all clusters to be more relevant compared to Saudi pharmacists. This might reflect their practice experience in their home countries, especially as the majority, particularly those from Eastern Mediterranean countries, must practise locally for at least two years before they are allowed to travel and practise in Saudi Arabia.^21^^,^^40^ However, the analysis of not relevant responses showed observed significant non-relevancy associated with non-Saudi pharmacists for behaviours within the following competency groups: ‘patient consultation and diagnosis’, ‘budget and reimbursement’, ‘human resources and management’, and ‘procurement’. Non-relevance in the behaviours of the ‘patient consultation and diagnosis’ competency may have been perceived because the initial education of non-Saudis included little or no pharmacy practice or clinical-based learning to inform their practice equivalent to the local PharmD curricula, the dominant curricula currently, where pharmacy practice and clinical pharmacy courses and training are heavily weighted.^21^ Moreover, the significant non-relevancy observed in ‘budget and reimbursement’, human resources and management, and ‘procurement’ competency groups was associated significantly with male non-Saudi pharmacists. This may indicate that Saudi pharmacists primarily hold management and leadership roles. However, a comparison between male and female Saudi pharmacists showed higher relevance to OM competencies in favour of male pharmacists (78.87% male to 67.81% female). Therefore, further study on the impact of gender on leadership and management roles in pharmacy is suggested.

Considering the Saudi context, where almost 65% of pharmacists are non- Saudi and 81% of pharmacists are male, the findings of this study suggest a variation in competencies and of professional performance between Saudi and non- Saudi on the one hand and between male and female pharmacists on the other hand. Importantly, this finding indicates that sitting a licensure exam where merely minimum knowledge is measured to permit registration might not be enough. The development of a national competency framework with the core competencies required for performance excellence could provide a means to reduce the degree of variation shown in this study. It will additionally inform the development of competency-based education, not only to achieve the desired competencies for local pharmacists, but also of a national assessment and training programme for overseas pharmacists in order to establish a consistent performance before undertaking the licensure exam. Moreover, the availability of a competency framework would indeed inform the licensure exam and provide the foundations for new continuing education and continuing professional development activities.^17^ It would also support practising pharmacists to identify learning needs to attain and maintain fitness to practise.

Currently, where the Saudi government is implementing major modernisation reforms, including in the pharmacy sector, developing a national competency framework that meets the government's plans for an expansion of pharmaceutical care roles in community pharmacies and the localisation of pharmacists would be of paramount importance in terms of fulfilling the government's 2030 Vision plans.^41^^,^^42^ Addressing the country's needs for a qualified and competent pharmacy workforce to provide the new expanded roles in community settings requires education to be developed with respect to the needs-based competencies. By doing so, a huge reserve of local pharmacists will be equipped with the appropriate learning and competencies to meet the government plans.^43^

The methods employed in this study provide a replicable example to other regional or global pharmacy practice researchers on how the FIP GbCF can be used as a first step to develop a country-specific competency framework. However, specific attention should be paid to local contexts, as local healthcare needs and other priorities will determine the competencies required for equipping pharmacists to meet local needs.

As an ideal first step to develop a country-level competency framework, the quantitative and qualitative findings from the study will serve as a basis to develop a national competency framework for foundation level pharmacists in Saudi Arabia. Future work involves conducting an expert panel to establish competency framework development based on local healthcare needs, followed by a validation of the draft framework.

### 4.1 Limitations

This study has some limitations. The utilisation of the snowballing sampling approach and the low sample size may limit the generalizability of the findings. This might have been caused by the large size of the survey (109 questions) or it may reflect a limited understanding of the concept and of the importance of competencies in professional development. Despite that, the number of responses in this study is comparable to other similar studies conducted elsewhere. ^6^^,^^17,28,31,32^ In addition, the low representation of academia, pharmaceutical industry and regulatory organisations may not provide a clear picture about their professional practice nor competencies required to fulfil their roles. Moreover, the lack of a specific definition of foundation-level pharmacy practice in Saudi Arabia may not provide a clear picture about the progress of pharmacists' performance over years and the relevance of the FIP GbCF to the practice of the foundation level pharmacists in the country. Additionally, the use of a self-completed survey may lead to self-reporting bias by the respondents. The use of multiple reminders may have led to duplicated responses, particularly given that the survey platform did not provide a feature to prevent this. Therefore, the results should be interpreted with caution.

## 5 Conclusion

This study investigated the relevance of the FIP GbCF in the Saudi pharmacy practice environment. The study found that competencies included in the GbCF v1 are relevant to pharmacy practice in the study population. However, some competencies and behaviours of the GbCF v1 require modification to be appropriate for the local needs of Saudi pharmacy practice and to develop a country-specific competency framework. The findings from this exercise will be used as a base to develop a foundation-level competency framework to inform the pharmacy education and professional development process in Saudi Arabia, equipping pharmacists to best meet local healthcare needs.

## Declaration of Competing Interest

The authors declare that they have no known competing financial interests or personal relationships that could have appeared to influence the work reported in this paper.
